# Optimizing *de novo* assembly of short-read RNA-seq data for phylogenomics

**DOI:** 10.1186/1471-2164-14-328

**Published:** 2013-05-14

**Authors:** Ya Yang, Stephen A Smith

**Affiliations:** 1 Department of Ecology & Evolutionary Biology, University of Michigan, 830 North University Ave, Ann Arbor, MI 48109-1048, USA

**Keywords:** 1KP, Chimera, *De novo* assembly, Redundancy, RNA-seq, SOAPdenovo-Trans, Trans-ABySS, Transcriptome, Trinity, Oases

## Abstract

**Background:**

RNA-seq has shown huge potential for phylogenomic inferences in non-model organisms. However, error, incompleteness, and redundant assembled transcripts for each gene in *de novo* assembly of short reads cause noise in analyses and a large amount of missing data in the aligned matrix. To address these problems, we compare *de novo* assemblies of paired end 90 bp RNA-seq reads using Oases, Trinity, Trans-ABySS and SOAPdenovo-Trans to transcripts from genome annotation of the model plant *Ricinus communis*. By doing so we evaluate strategies for optimizing total gene coverage and minimizing assembly chimeras and redundancy.

**Results:**

We found that the frequency and structure of chimeras vary dramatically among different software packages. The differences were largely due to the number of *trans*-self chimeras that contain repeats in the opposite direction. More than half of the total chimeras in Oases and Trinity were *trans*-self chimeras. Within each package, we found a trade-off between maximizing reference coverage and minimizing redundancy and chimera rate. In order to reduce redundancy, we investigated three methods: 1) using cap3 and CD-HIT-EST to combine highly similar transcripts, 2) only retaining the transcript with the highest read coverage, or removing the transcript with the lowest read coverage for each subcomponent in Trinity, and 3) filtering Oases single *k*-mer assemblies by number of transcripts per locus and relative transcript length, and then finding the transcript with the highest read coverage. We then utilized results from blastx against model protein sequences to effectively remove *trans* chimeras. After optimization, seven assembly strategies among all four packages successfully assembled 42.9–47.1% of reference genes to more than 200 bp, with a chimera rate of 0.92–2.21%, and on average 1.8–3.1 transcripts per reference gene assembled.

**Conclusions:**

With rapidly improving sequencing and assembly tools, our study provides a framework to benchmark and optimize performance before choosing tools or parameter combinations for analyzing short-read RNA-seq data. Our study demonstrates that choice of assembly package, *k*-mer sizes, post-assembly redundancy-reduction and chimera cleanup, and strand-specific RNA-seq library preparation and assembly dramatically improves gene coverage by non-redundant and non-chimeric transcripts that are optimized for downstream phylogenomic analyses.

## Background

With the recent and rapid advance of sequencing techniques, transcriptome sequencing (RNA-seq) has emerged as a powerful tool for obtaining large amount of functional genomic data in non-model organisms. This has encouraged efforts such as the One Thousand Plants Project, or 1KP [1], and many other transcriptome projects. Each of these data sets contains sequence information for thousands of genes, showing huge potential for phylogenomic inference. However, there are many analytical and computational challenges that come with analyzing these data sets. One of the biggest challenges is to accurately assemble the short reads from non-model organisms that do not have any reference genome, or *de novo* transcriptome assembly. Because this is the first step in any phylogenomic analysis, problems at this stage (incomplete assembly, assembly errors, and redundancy) cause difficulties for downstream analyses including ortholog and paralog identification, alignment, and matrix construction. These problems increase the amount of missing data in the final aligned matrix, ultimately limiting the amount of useful transcriptomic data for phylogenomics.

Currently, the most popular software packages for short-read RNA-seq assembly include Oases [2,3], Trinity [4], trans-ABySS [5,6] and SOAPdenovo (to be replaced by SOAPdenovo-Trans for transcriptome assembly [7]). All four packages are based on constructing, simplifying, and resolving de Bruijn graphs to extract likely transcripts (see [8] for a general introduction). Two of these, Oases [2,3] and trans-ABySS [5,6] start with constructing de Bruijn graphs directly from sequencing reads, remove potential errors, and then resolve each de Bruijn graph to extract transcripts for each connected component (i.e. cluster, or “locus”) in the graph. Both packages use a range of *k*-mer sizes to accommodate variation in read coverages among genes. Trinity [4], on the other hand, uses a single *k*-mer with size fixed at 25 bp. Trinity first carries out a greedy extension step starting from the most abundant *k*-mer to build linear contigs, groups overlapping contigs into connected components, and constructs a de Bruijn graph for each component. Sequencing reads are then mapped to the graphs, the graphs are simplified, errors are removed (which may break a component into subcomponents), and finally likely isoforms are extracted for each component or subcomponent. All four also use the information from mate pairs to assemble contigs into scaffolds when paired end reads are available. Each “locus” from the Oases output (roughly equivalent to component/subcomponent in Trinity) consists of one or more “transcripts” (or “isoforms” in Trinity) [2,4]. Biologically a locus or a component/subcomponent can each contain one gene or several paralogs, and a single gene can have fragments distributed among multiple loci or components/subcomponents [2]. Trans-ABySS does not explicitly output sequences in hierarchical groups. SOAPdenovo-Trans is available only as precompiled executables without formal publication or source code [7]. Therefore we are unable to evaluate its assembly method in detail, and only include it here for completeness.

All the published *de novo* transcriptome assemblers are optimized for building references for comparing gene expression levels, identifying splice variants, and determining gene fusion events [2,4,5]. For phylogenomic purposes, however, only one representative transcript for each gene is required. Splice variants are not only unutilized but also complicate the detection of true paralogs for phylogenomic matrix construction. One method for picking the highest covered transcript from a locus from Oases assemblies includes choosing the isoform with the highest geometric mean read coverage across nodes [9]. By using geometric mean, regions of very low expression that may be associated with assembly error were penalized. Another way of selecting isoforms, as suggested by the Trinity documentation [10], is by mapping sequencing reads back to the assembled transcripts, and either picking the isoform with the highest coverage, or removing the isoform with the lowest coverage in a subcomponent. However, strategies for picking the dominant and correct transcript have not been extensively explored.

**Figure 1:**
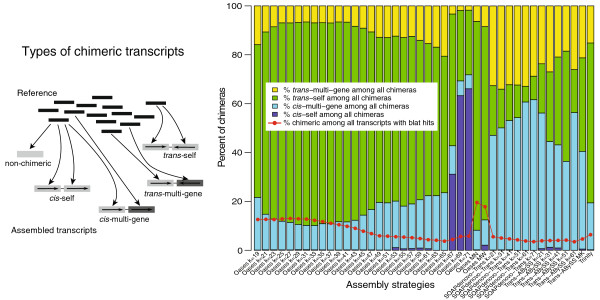
**Chimera compositions among assembled transcripts before post-processing.** Oases MN: Oases-M merging single *k*-mer assemblies of 21, 31, 41, 51 and 61; MW: Oases-M merging single *k*-mer assemblies of 19–71, with increment of 2; Trans-ABySS MK: Trans-ABySS merging single *k*-mer assemblies of 21, 31, 41, 51 and 61.

A second problem that has been largely ignored by previous *de novo* transcriptome analyses is the creation of chimeras. Only “fusion transcripts” formed from multiple genes have been discussed in previous studies without further discussion of their nature and sources [4,11-13]. Chimeras can come from misassembly of short reads or PCR-induced recombination during library preparation. Chimeras may also be real biological products from gene fusion or *trans-*splicing. In the case of short read assemblies, it is reasonable to assume that mis-assembly is the predominant cause, and by comparing different *de novo* assembly strategies, the difference is mainly caused by the assembly process. In this study, by “chimera” we are referring to structural anomalies caused by all three potential sources. If a chimera contains the same gene repeating itself, it is a self chimera. If a chimera is the result of multiple genes, it is a multi-gene chimera. If the sequences are assembled together in the same direction, it is considered to be a *cis* chimera. If the sequences are assembled in opposite directions, it is a *trans* chimera. Considering both gene composition and direction, we recognized four types of chimerisms: *cis*-self, *cis*-multi-gene, *trans-*self and *trans-*multi-gene (Figure 1). Recognizing these four types of chimeras helps to identify the potential causes of misassemblies, and enables us to design effective post-assembly filters for removing chimeras.

Here, we examine the extent of problems with chimeras as well as redundancy in *de novo* transcriptome assembly with the goal of optimizing transcript choice for phylogenomic analyses. The castor bean (*Ricinus communis*, Euphorbiaceae) provides a unique opportunity for addressing these problems because it allows for direct comparison of a Sanger sequencing-derived draft genome sequence [14] with a typical *de novo* assembly of 90 bp paired end RNA-seq data, recently generated as part of the 1KP project [15]. Here we compare assemblies from Oases, Trinity, Trans-ABySS and SOAPdenovo-Trans to the genome-derived gene annotations of *R. communis*, to evaluate assembly strategies and post-assembly filters that best reduce redundancy while simultaneously maximizing total gene coverage and minimizing assembly chimeras.

## Methods

### Data set

Short-read RNA-seq results were obtained from the 1KP project database. Sampling and voucher information is available from the 1KP website (sample id: PAZJ) [1]. Procedures of RNA extraction, quality control, and library construction are detailed in Johnson et al. [15]. In summary, RNA was obtained from mixed sample of leaves and flower buds. Sequencing was carried out on the Illumina HiSeq2000 platform, with paired end 90 bp reads and insert size of around 200 bp.

Non-redundant transcripts from genome annotation of *R. communis* were downloaded from Phytozome v. 9.0 [16]. *R. communis* chloroplast [GenBank:JF937588] and mitochondrial [GenBank:HQ874649] genome sequences were downloaded from the GenBank, and together they constitute the organellar genome sequences.

### Sequence cleanup and *de novo* assembly

Downloaded reads were subjected to the following cleaning steps: (1) read pairs were removed if either of the reads in the read pair had average quality score ≤ 32; (2) read 3’ end nucleotides with quality scores < 10 were trimmed, and post-trim read pairs with either of the reads ≤ 72 bp were removed; and (3) reads with adaptor contamination were removed.

Possible chloroplast operons were observed when attempting *de novo* assembly with all reads, creating enormously complex graphs with thousands of assembled transcripts per locus in Oases. No mitochondrial operons were observed. In order to reduce complexity and separate naturally occurring multi-gene transcripts from chimeras induced by assembly errors, cleaned read pairs that concordantly mapped to *R. communis* organellar genomes were removed using Bowtie 2 v. 2.0.0-beta7 [17].

Cleaned and filtered nuclear reads were assembled *de novo* using four software packages. (1) Trinity v. 2012-02-25 [4] was used with the default settings including a fixed *k*-mer size of 25 as suggested by the authors. (2) Oases v. 0.2.08 [2] was used with *k*-mer sizes from 19 to 71, with an increment of 2 and an average insert length 200 bp. The Oases-M pipeline was used to merge assemblies from single *k-*mer Oases assemblies either with all transcripts from *k* = 19 to 71 (Oases-M-wide-range, or MW), or with a narrower range of *k* = 21, 31, 41, 51 and 61 (Oases-M-narrow-range, or MN). (3) ABySS v. 1.3.4 [6] was used with *k*-mer sizes 21, 31, 41, 51 and 61, and contigs were filtered, extended and merged using Trans-ABySS v. 1.4.4 [5]. (4) SOAPdenovo-Trans v. 1.01 [7] was used with *k*-mer sizes 21, 31, 41, 51, 61 and 71 with default settings. Only assembled transcripts longer than 200 bp were kept.

### Evaluating *de novo* assembly by comparing to the reference transcripts

Downloaded non-redundant reference transcripts from *R. communis* genome annotation were compared to the *R. communis* organellar genome sequences using default blat settings [18]. Transcripts with ≥ 80% combined region that mapped with ≥ 95% similarity to organellar genome sequences were removed. The remaining nuclear transcripts were used as reference for accessing the quality of *de novo* assemblies of nuclear reads.

Results from *de novo* assembly were mapped to the reference nuclear transcripts using default blat settings [18]. Blat hits were processed in three steps. First, we ignored hits with lower than 95% similarity or less than 100 matching base pairs. Next, if an assembled transcript had only one blat hit, it was considered to be the best hit, and the assembled transcript was considered to be non-chimeric. Finally, if an assembled transcript had more than one blat hits, the best hit with the highest number of matching nucleotides was found. The assembled transcript was identified as a chimera if any of the rest blat hits had no, or minimal, overlap in query coverage to the best hit (overlap less than 60 bp and less than 20% of the shorter of the pair). If none of these comparisons identified chimeras, the longest hit was considered the best hit, and the assembled transcript was identified as non-chimeric.

### Selecting highly-covered isoforms for reducing redundancy

All four *de novo* assembly packages output large numbers of sequence isoforms, some of which are real splice variants, but many are chimeras. For phylogenomic analysis, only a single representative, correct transcript is desired. We investigated, in more detail, strategies to choose the representative isoforms in a multiple-*k* package Oases, and in a single-*k* package Trinity, since these two packages group assembled sequences into either locus or component/subcomponent explicitly in their output. Both also have relatively detailed documentations for their algorithms [2-4,10,19].

In Oases, very short sequence motifs in a locus tend to have high read coverage and need to be filtered out first. We tested only considering transcripts longer than 0.3, 0.5, 0.7 or 0.85 of the longest transcripts in the same locus. After filtering transcripts by their relative lengths, the remaining transcript with the highest read coverage as measured by the geometric mean of read coverages across nodes was chosen as the representative transcript. We then calculated the percentage of the representative transcripts being chimeric vs. *k-*mer size, and number of transcripts per locus.

By examining percent chimera vs. relative length filter, *k-*mer size, and number of transcripts per locus plots, we applied a relative length filter of 0.3, and chose the representative transcripts from *k* = 21, 31, 41, 51 only when there was 1 transcripts per locus, with no limit on number of transcripts per locus at *k* = 61 (filter1). With this parameterization, gene families would only be represented at relatively high values of *k*. In addition of filter1, we also tested retaining the representative transcripts from *k* = 21, 31, 41, 51 when there was either 1 or 3 transcripts per locus with no limit on number of transcripts per locus at *k* = 61 (filter1&3). The program cap3 [20] was then used to merge exemplars with overlap length cutoff of 200 and overlap percent identity cutoff of 99 (−o 200 -p 99). We also tested using CD-HIT-EST [21] instead of cap3 for removing redundancy with sequence identity cutoff set to 0.98 (−c 0.98 -n 10 -r 1).

To identify the representative isoforms in Trinity, we mapped reads to the assembled transcripts using RSEM [22], and either only retained the isoforms with the highest isoform percentage (IsoPct) within each subcomponent (Trinity-pickH), or removed the isoforms with the lowest IsoPct if there were more than one isoforms per subcomponent (Trinity-removeL).

Trans-ABySS does not explicitly group assembled transcripts by graph component. Therefore we were unable to investigate strategies of choosing the dominant isoforms for each component other than by merging sequences by similarity. Trans-ABySS multiple-*k* results were subject to cap3 (−o 200 -p 99) to combine similar sequences.

No multiple-*k* pipeline was provided with the current release 1.01 of SOAPdenovo-Trans. To explore the effect of combining multiple *k*-mers in SOAPdenovo-Trans, we combined all scaffolds from SOAPdenovo-Trans *k* = 21, 31, 41, 51 and 61 using cap3 (−o 200 -p 99). Alternatively, we tested combining all contigs of *k* = 21, 31, 41, 51 and 61 instead of scaffolds, since the Ns inserted in the scaffolds interfere with setting a similarity score cutoff for combining.

### Post-assembly trans chimera removal without reference

Assembled sequences were blasted against non-redundant peptide sequences from 26 model eudicot species downloaded from the Phytozome v. 9.0 database [16], excluding *R. communis*. The closest relative to *R. communis* in the database was *Manihot esculenta*, which split from the lineage leading to *Ricinus* approximately 85 million years ago [23]. In order to facilitate chimera identification, blastx was carried out with a relatively high e-value cutoff of 0.01, and max_target_seqs set to 100.

*Trans* chimeras were detected from blastx results with three steps. First, High-scoring Segment Pairs (HSPs) with less than 30% identity or with query coverages shorter than 100 bp were ignored. Second, *trans-*self chimeras were detected from HSPs between a query-target pair. Detection of *trans-*self chimeras (Figure 1, left side) included the following steps:

1) Since HSPs of the same direction were most often separated in the blastx results due to indels from sequencing errors, we considered all the query coverages in the same direction represented one single ORF. If all HSPs from a query-target pair were in the same direction, we ignored all subsequent HSPs and continued to the next query-target pair.

2) If HSPs between a query-target pair were in the opposite directions, we merged all query coverages of plus direction, and merged all query coverages of minus direction.

3) If the overlap between the merged plus and minus query coverages were less than 60 bp and less than 20% of the shorter one of the two, the sequence was labeled as *trans*-self chimera, and the transcript was cut leaving only the longer of the merged query coverages. All subsequent HSPs from the same query were ignored.

Finally, if no *trans*-self chimera was detected, we continued to check for *trans-*multi-gene chimeras (Figure 1, left side) from all HSPs of the same query against different targets. We did this with the following steps:

1) All query coverages of plus direction were merged. Similarly, all query coverages of minus direction were merged.

2) If the overlap between the merged plus and minus query coverages from the same query were less than 20% of the shorter one of the two and less than 60 bp, the query sequence was cut leaving only the longer query coverage. Chimeras detected at this step are of the *trans*-multi-gene type. This is because those that have different regions hitting the same target were detected as *trans*-self chimeras at the previous step and the query range being cut out. In this case the *trans*-multi-gene detection steps were skipped.

Only sequences that were longer than 200 bp after cutting were retained. No *cis* chimeras were removed. This is because when blasting to distantly related model species, tandem duplication, rearrangement, and heterogeneity in evolutionary rates among segments within a gene could result in the false detection of *cis* chimeras.

### Calculating total reference coverage and redundancy

For each assembly strategy tested, only the best blat hit from non-chimeric sequences was used for calculating total reference coverage. For each nuclear reference transcript, only the longest non-chimeric reference coverage from blat hits was used to calculate the total coverage, as downstream phylogenomic analyses will only use one sequence per gene. Final reference coverage from each assembly strategy was calculated by percentage of total reference base pairs assembled to longer than 200 bp, percentage of reference genes assembled to longer than 200 bp, and percentage of reference genes assembled to 80% or more. We measured redundancy as the number of assembled sequences with blat hits divided by the number of reference transcripts with blat hits.

All scripts used in the methods are available from Bitbucket [https://bitbucket.org/yangya/optimize_assembler].

## Results and discussion

### *De novo* assembly of short RNA-seq reads recovered up to half of total genes

The RNA-seq data set consisted of 11,041,065 read pairs. Of these, 9,527,760 (86.3%) met our quality criteria and were free of adaptor contamination. Organellar reads accounted for a third of the cleaned reads, leaving 6,220,964 (65.3%) nuclear reads. Of all the cleaned and filtered nuclear reads, 95.1% retained their original read length of 90 bp. The remaining ranged from 73 to 89 bp in length (Additional file 1).

Out of the total 31,221 annotated nuclear and organellar genes in *R. communis*, 30,743 (98.5%) were nuclear. Comparison of *de novo* assembly of nuclear reads to reference nuclear transcripts shows that up to 14,539 (47.3%, Oases-MW) of these were assembled to longer than 200 bp with *de novo* methods. This confirms that *de novo* assembly of short read RNA-seq data is capable of recovering close to half of the genes in a nuclear genome with mixed tissue types of leaves and flower buds. This number could be further increased with deeper sequencing depth or a higher diversity of tissue types. Such high gene coverage demonstrates the huge potential of RNA-seq data in obtaining exome sequences in non-model organisms. However, it also raises the question of why many phylogenomic analyses that use short-read RNA-seq data only include hundreds of genes [9,24-26], instead of thousands or even tens of thousands of genes, as in similar studies that incorporate longer reads from Sanger or 454 sequencing [27,28]. Close scrutiny of assembly and post-assembly cleanups are the first steps towards increasing the matrix occupancy in phylogenomic analyses.

### Types and frequencies of chimeras vary dramatically among assembly strategies

Our chimera detection criteria differ from previous studies in taking self chimeras into account and allowing short overlap between query coverages. These detection criteria enabled us to thoroughly examine a wider range of chimeras.

Among the four assembly packages, overall percentages of chimeras (Figure 1, red line; Additional file 2) were lowest among Trans-ABySS assemblies (0.81–2.01%). Oases assemblies, both from single *k*-mer (1.1–11.0%) and multiple *k*-mer (18.0% from narrow *k* range MN and 16.1% from wide *k* range MW), produced some of the highest percentages of chimeras.

The relationship between chimera rate and *k*-mer size varied among the three multiple-*k* assembly packages (Figure 1 and Additional file 2). Among single *k*-mer assemblies in Oases, the percentage of chimeras was lowest when *k* = 65 (1.1%), and increased towards both lower and higher *k*. The trend among Trans-ABySS single *k*-mer assemblies was opposite, with the highest percentage of chimera at *k* = 51 (1.52%), and decreased towards both lower and higher *k*. As for SOAPdenovo-Trans, percentages of chimeras decreased from 2.99% when *k* = 21 to 0.85% when *k* = 71. Both default multiple-*k* assembly pipelines by Oases and Trans-ABySS produced higher percentages of chimeras as compared to single *k*-mer assemblies using the same software packages.

The composition of chimeras also varied dramatically among different assembly strategies. The majority of chimeras produced by Oases and Trinity were *trans*-self (Figure 1, green), except in Oases single-*k* assemblies when *k* was very large (67, 69 and 71). Vast majority of all chimeras produced by SOAPdenovo-Trans were either *trans*- or *cis*-multi-gene chimeras. All four types of chimeras except *cis*-self had roughly equal share among assemblies using Trans-ABySS. The difference in overall percentage of chimeras among assembly methods was largely contributed by the drastic difference in *trans*-self chimeras (Figure 1 and Additional file 2). Such drastic differences may relate to how contigs are assembled into scaffolds and how hairpin loops in the de Bruijn graph are resolved [19]. However, so far very little discussion is available on how loops in de Bruijn graphs should be resolved, and whether different approaches should be taken between genome (where repeats are expected) vs. transcriptome assemblies (where frequencies of repeats differ between coding and non-coding regions).

The analyses presented here suggest a few general conclusions about transcriptomic assemblies. 1) The number and composition of chimeras differ dramatically among different assembly strategies, and the difference is largely due to the number of *trans*-self chimeras. 2) Higher *k*-mer size does not necessarily lead to a smaller percentage of chimeras. 3) Merging multiple single *k*-mer assemblies increases the percentage of chimeras. With a chimera rate of at least 16%, Oases-M, including both narrow and wide range of *k*, is not suitable for downstream analyses.

### Strategies for choosing highly covered isoforms to reduce redundancy in Oases

Another important consideration, especially for phylogenomics, is redundancy in assembly results. Each component/subcomponent in the Trinity output, or each locus in Oases, includes fragments of a gene or clusters of paralogous genes [2,10], and often contains splice variants and/or chimeras.

**Figure 2:**
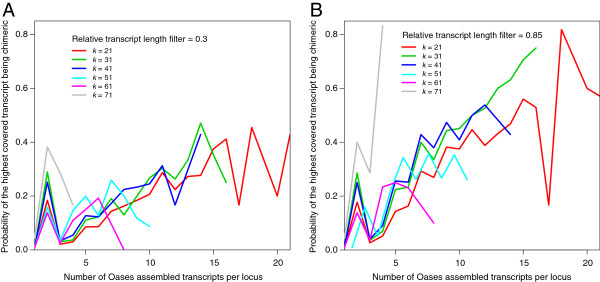
**Parameters for choosing the representative transcript for each locus in Oases.** (**A**) Only considering transcripts that are longer than 0.3 of the longest transcript in the same locus; (**B**) only considering transcripts that are longer than 0.85 of the longest transcript in the same locus. A data point is plotted only when there are five or more loci of the same size in each data set.

In Oases, very short transcripts that represent conserved motifs often have much higher read coverage compared to longer transcripts in the same locus. These very short transcripts need to be filtered out before selecting exemplars. We examined subsets of transcripts with lengths longer than 0.3, 0.5, 0.7, and 0.85 of the longest transcript within the same locus respectively. We found that with a higher proportion cutoff, it was more likely that the dominant transcript was a chimera (Figure 2, showing relative transcript length filter 0.3 vs. 0.85; 0.5 and 0.7 not shown). Regardless of the value of *k*, loci that had high numbers of transcripts were likely to contain many chimeras. The probability of the highest covered transcript being chimeric generally increased with size of the locus to as high as 0.83 (Figure 2b, *k* = 71). However, the probability remained relatively low (≤ 0.05) when size of locus was equal to 1 or equal to 3. The spike of chimeras at two transcripts per locus (Figure 2) was almost exclusively caused by change in *trans*-self chimeras.

In Oases, chimera rate decreased with increasing *k* until *k* = 65. This was largely a result of the production of smaller loci with fewer numbers of transcripts per locus as *k* increased. Therefore, by only including loci with either one transcript (filter1), or one and three transcripts (filter1&3), the vast majority of chimeras were excluded. To preserve members of gene families and alternatively-spliced genes, we included all transcripts at *k* = 61 regardless of number of transcripts per locus. In this way, we only retained assemblies of these genes at a relatively high *k-*mer size when each locus had fewer transcripts, and therefore less likely to produce chimeras. Although we only investigated filtering loci by numbers of transcripts per locus and *k-*mer sizes in Oases, similar methods are potentially useful for other software packages using multiple*-k* assembly strategies*.*

### Post-assembly *trans* chimera removal by blastx

After assembly, a blastx analysis of the transcripts against known protein sequences is a routine step for quality checking and downstream analyses such as homolog clustering and functional annotation. Here we take advantage of results from blastx to detect chimeras as well. *Cis* chimeras cannot be reliably detected when compared to sequences in a related species. Tandem duplication and rearrangement of gene segments can cause a false identification of *cis*-self chimera, and heterogeneity in base pair substitution rate within a gene can produce blastx hits similar to *cis*-multi-gene chimera. *Trans* chimeras, on the other hand, are much easier to detect from blastx results. In the majority of eukaryotic nuclear genomes, a transcript is unlikely to have two different ORFs of the opposite direction, especially if each of these ORFs is highly similar to known coding sequences, of sufficient length, and there is no substantial overlap between these ORFs.

**Figure 3:**
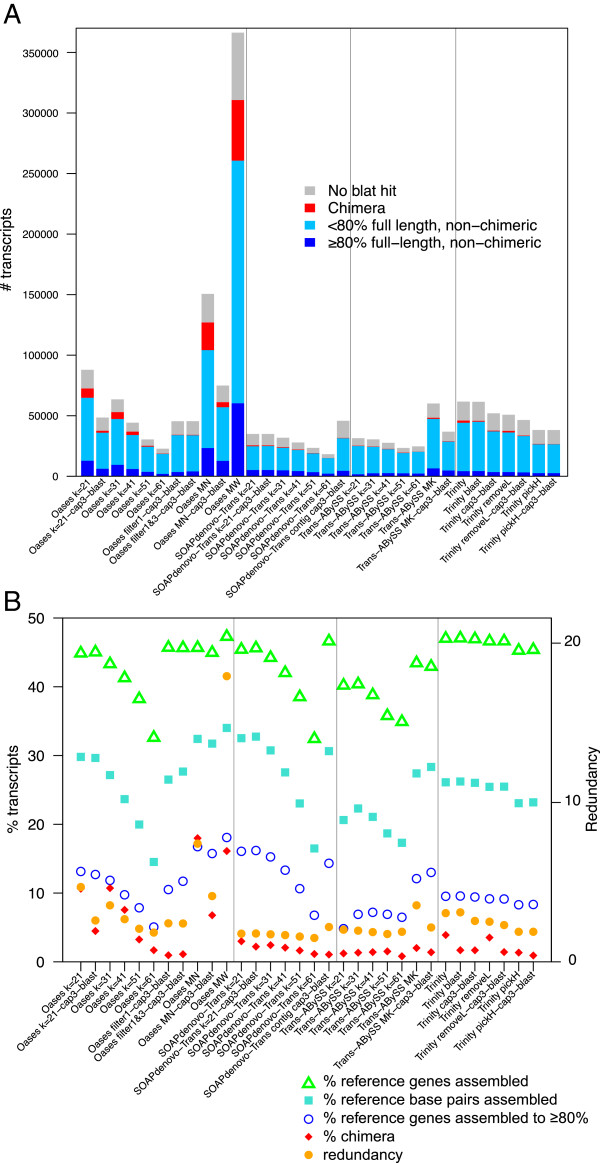
**Overall comparison among assembly strategies.** (**A**) Number of transcripts in each category; and (**B**) percent reference coverage, redundancy and chimera rate among assembly strategies. Cap3: redundancy reduction using cap3; blast: *trans* chimera cleanup using blastx against model protein database; Oases MK filter: filter loci from Oases single *k*-mer assemblies by number of transcripts per locus at *k* = 21, 31, 41 and 51, with *k* = 61 not subject to filtering by number of transcripts per locus, before combining them. Oases MN: Oases-M merging single *k*-mer assemblies of 21, 31, 41, 51 and 61; MW: Oases-M merging single *k*-mer assemblies of 19–71, with increment of 2; SOAPdenovo-Trans contigs: combining contigs from SOAPdenovo-Trans single *k*-mer assemblies of 21, 31, 41, 51 and 61; Trans-ABySS MK: Trans-ABySS merging single *k*-mer assemblies of 21, 31, 41, 51 and 61; Trinity pickH: only keeping the transcript with the highest read coverage for each subcomponent; Trinity removeL: when there are two or more transcripts per subcomponent, remove the one with the lowest read coverage.

Our results regarding chimera removal were most successful in Oases and Trinity, both having *trans* chimeras as dominant chimera types. The percentage of chimeras in the Oases-MN assembly were reduced from 18.0% to 6.8% after using cap3 to merge similar reads and subsequent blastx and cutting of *trans* chimeras (Figure 3, Additional file 3). Similarly, chimeras were reduced from 10.6% to 4.5% in Oases *k* = 21, and from 3.9% to 1.7% in Trinity. Chimera rates were reduced from 2.0% to 1.4% in Trans-ABySS MK, and from 3.0% to 2.2% in SOAPdenovo-Trans *k* = 21, where *trans* chimeras account for a relatively small percentage of total chimeras.

In addition to using results from blast against other model species, one can also detect chimeras by mapping reads back to assembled transcripts to identify areas with a sudden change in read coverage. However, this may not work for self chimeras. Methods have been developed for detecting fusion transcripts by mapping reads to a conspecific reference genome. How effective it is doing so using transcripts assembled *de novo* has not yet been explored.

### Overall comparison of gene coverage, chimera and redundancy

Composition of assembled transcripts, reference gene coverages, and redundancy are summarized in Figure 3 and Additional file 3. The best assembly strategies are those that maximize number and length of reference covered, measured as percent gene covered, percent base pairs covered, and percent genes assembled to >80% (green, turquoise and blue respectively in Figure 3b), yet with a low chimera rate and redundancy (Figure 3b, red and orange).

Overall, we found a trade-off between maximizing reference coverage vs. minimizing chimera rate and redundancy within each package before post processing using cap3 and blastx. With proper post processing, however, results from seven assembly strategies among all four assembly packages converged to very similar values: all seven successfully assembled 42.9–47.1% of reference genes to more than 200 bp, with a chimera rate of 0.92–2.21%, and 1.78–3.11 transcripts per reference gene assembled (Table 1).

**Table 1:** Summary statistics among seven highest performing assembly strategies

**Assembly strategies**	**Percent reference coverage (bp)**	**Percent reference genes assembled to >200 bp**	**Percent reference genes assembled to ≥80%**	**Percent chimeric**	**Redundancy**
Oases filter1-cap3-blast	26.50%	45.70%	10.52%	0.96%	2.42
Oases filter1&3-cap3-blast	27.67%	45.65%	11.73%	1.11%	2.41
SOAPdenovo-Trans *k*=21-cap3-blast	**32.75%**	45.62%	**16.20%**	2.21%	**1.78**
SOAPdenovo-Trans contigs cap3-blast	30.64%	46.62%	14.34%	1.06%	2.19
Trans-ABySS MK-cap3-blast	28.34%	42.93%	13.01%	1.39%	2.16
Trinity blast	26.22%	**47.06%**	9.58%	1.69%	3.11
Trinity pickH-cap-blast	23.19%	45.38%	8.35%	**0.92%**	1.89

The best score for each column, measured as highest in reference coverage and lowest in chimera rate and redundancy, is in bold.

Among Oases assemblies (Figure 3 and Additional file 3), while single *k* (*k* = 41, 51 and 61) assemblies suffered from low reference coverages, Oases single *k* (*k =* 21 and 31) and Oases MN, MW assemblies all suffered from relatively high chimera rates and redundancies. The problems with high chimera rates and redundancies persisted even after using cap3 to reduce redundancy and blastx to remove *trans* chimeras in two of the strategies with relatively high reference coverages (*k* = 21 and MN). We also tested using CD-HIT-EST instead of cap3 to remove redundancy, and the outcome was very similar to cap3 results (data not shown). The default Oases-M pipeline takes transcripts from single*-k* assemblies as input and assembles them using *k* = 27. This strategy is one step removed from the original read coverage information, and keeps most isoforms and errors from the original single *k*-mer assemblies. Our two novel Oases MK pipelines “Oases filter1-cap3-blast” and “Oases filter1&3-cap3-blast” utilize the read coverage and number of transcripts per locus information in single *k*-mer assemblies, and achieve both low redundancy and low chimera rates with high reference coverages (Table 1 and Figure 3).

All Trans-ABySS single *k*-mer assemblies suffered from relatively low reference coverages (Figure 3 and Additional file 3), whereas Trans-ABySS MK, especially after post processing, ranks among one of the highest performing assembly strategies tested (Table 1). However, Trans-ABySS, as a package, carries out many functions beyond sequence assembly without detailed documentation. The current version 1.4.4 is not as user-friendly as the other packages tested in this study.

Trinity, Trinity blast, and Trinity cap3-blast are the top three assembly strategies in terms of total reference genes assembled (Additional file 3). Between Trinity blast and Trinity cap3-blast, adding a cap3 step reduced redundancy from 3.1 to 2.6, while slightly reduced reference coverage. An alternative approach of reducing redundancy by removing the lowest covered transcript in a subcomponent (Trinity removeL) reduced both redundancy and chimera rate only slightly, at a cost of slightly reducing reference coverage as well. This suggests that the lowest covered transcripts can be either chimeras or paralogous genes that have relatively low expression levels. Similarly, only keeping the highest covered transcripts for each subcomponent (Trinity pickH) further reduced reference coverage, redundancy, and chimera rate. This strategy is a more aggressive way of reducing redundancy than cap3 alone or removeL. However, since Trinity started with the highest reference coverage by the number of genes, after pickH combined with cap3 and blastx, this very aggressive post processing strategy produced one of the cleanest, lease redundant assemblies, and the overall reference coverage is not very far below the other high performing assembly strategies (Table 1).

SOAPdenovo-Trans *k*=21-cap3-blast produced the highest reference coverage measured by base pairs only after Oases MW (Table 1 and Additional file 3: Table S3). It does this by aggressively scaffolding from contigs, and this process more than doubled the percentages of chimeras (Additional file 2: Table S2). The Ns inserted in many of the scaffolds also made it difficult to find a suitable similarity cutoff for merging scaffolds from multiple single *k*-mer SOAPdenovo-Trans assemblies using cap3. Instead of using scaffolds, we merged all contigs from SOAPdenovo-Trans *k* = 21, 31, 41, 51 and 61 using cap3. This strategy, SOAPdenovo-Trans contigs cap3-blast, turns out to be among the highest performing strategies tested (Table 1), comparable to or outperforms most strategies incorporating mate pair information. This suggests that with more accurate scaffolding, SOAPdenovo-Trans has the potential to further improve its performance.

In summary, in our particular data set we identified seven strategies among four assembly packages that produced results that are most suitable for downstream phylogenomic analysis. All seven scored very close to each other in reference coverage, chimera rate, and redundancy, suggesting that the most suitable assembly strategy can vary with different data sets, and that there is no single best strategy for all assembly tasks.

## Conclusions

*De novo* assembly of short read RNA-seq data is capable of recovering up to half of the total expressed genes to more than 200 bp. However, Oases, Trinity, and to a lesser extent Trans-ABySS, all produce large amounts of *trans*-self chimeras. We find that although Oases-M produces the highest gene coverage among popular assembly packages, its high redundancy and chimera rate make it unsuitable for phylogenomic purposes, even with extensive post processing. Trinity achieves high reference coverage that is similar to Oases-M, but with a much lower redundancy and chimera rate, especially after *trans* chimera removal using blastx and effective redundancy reduction. SOAPdenovo-Trans, although yet unpublished, is highly promising in producing some of the cleanest assemblies with the highest reference coverage.

Many packages used in next generation sequence analyses were initially optimized for purposes other than phylogenomics, and care should be taken when utilizing these packages for phylogenomics. With the rapid development of both sequencing techniques and software packages, one needs to examine the specific types and structures of assembly problems in order to minimize them. Future evaluation of *de novo* assembly tools for phylogenomics should focus on completeness [29], chimerism, and redundancy, instead of ambiguous measures borrowed from genome assembly, such as N50. Lastly, since a significant number of the chimeras are *trans-*chimeras, strand-specific library preparation and assembly, as demonstrated by Garg et al. [30], can eliminate a major source of assembly errors.

## Competing interests

The authors declare that they have no competing interests.

## Authors’ contributions

YY and SAS designed the study, carried out the analyses, drafted the manuscripts, and read and approved the final manuscript.

## Supplementary Material

Additional file 1 Length distribution of cleaned and filtered nuclear reads. Click here for file

Additional file 2 Chimera compositions among assembled transcripts before post-processing. Click here for file

Additional file 3 Reference coverages, chimera percentages and redundancies among assembly strategies. Click here for file
